# Left Ventricular Noncompaction: Anatomical Phenotype or Distinct Cardiomyopathy?

**DOI:** 10.1016/j.jacc.2016.08.054

**Published:** 2016-11-15

**Authors:** Jonathan R. Weir-McCall, Phey Ming Yeap, Carla Papagiorcopulo, Kerrie Fitzgerald, Stephen J. Gandy, Matthew Lambert, Jill J.F. Belch, Ian Cavin, Roberta Littleford, Jennifer A. Macfarlane, Shona Z. Matthew, R. Stephen Nicholas, Allan D. Struthers, Frank Sullivan, Shelley A. Waugh, Richard D. White, J. Graeme Houston

**Affiliations:** a Department of Cardiovascular and Diabetes Medicine, College of Medicine, University of Dundee, Dundee, United Kingdom; b NHS Tayside Medical Physics, Ninewells Hospital, Dundee, United Kingdom; c Department of Research and Innovation, North York General Hospital, University of Toronto, Toronto, Ontario, Canada; d Department of Clinical Radiology, University Hospital of Wales, United Kingdom

**Keywords:** anatomy, cardiomyopathy, diagnostic, left ventricular noncompaction, magnetic resonance imaging, BNP, B-type natriuretic peptide, CMR, cardiac magnetic resonance imaging, CVD, cardiovascular disease, LVEDV, left ventricular end-diastolic volume, LVEF, left ventricular ejection fraction, LVESV, left ventricular end-systolic volume, LVGFI, left ventricular global function index, LVMVR, left ventricular mass volume ratio, LVNC, left ventricular noncompaction, LVSV, left ventricular stroke volume

## Abstract

**Background:**

There is considerable overlap between left ventricular noncompaction (LVNC) and other cardiomyopathies. LVNC has been reported in up to 40% of the general population, raising questions about whether it is a distinct pathological entity, a remodeling epiphenomenon, or merely an anatomical phenotype.

**Objectives:**

The authors determined the prevalence and predictors of LVNC in a healthy population using 4 cardiac magnetic resonance imaging diagnostic criteria.

**Methods:**

Volunteers >40 years of age (N = 1,651) with no history of cardiovascular disease (CVD), a 10-year risk of CVD < 20%, and a B-type natriuretic peptide level greater than their gender-specific median underwent magnetic resonance imaging scan as part of the TASCFORCE (Tayside Screening for Cardiac Events) study. LVNC ratios were measured on the horizontal and vertical long axis cine sequences. All individuals with a noncompaction ratio of ≥2 underwent short axis systolic and diastolic LVNC ratio measurements, and quantification of noncompacted and compacted myocardial mass ratios. Those who met all 4 criteria were considered to have LVNC.

**Results:**

Of 1,480 participants analyzed, 219 (14.8%) met ≥1 diagnostic criterion for LVNC, 117 (7.9%) met 2 criteria, 63 (4.3%) met 3 criteria, and 19 (1.3%) met all 4 diagnostic criteria. There was no difference in demographic or allometric measures between those with and without LVNC. Long axis noncompaction ratios were the least specific, with current diagnostic criteria positive in 219 (14.8%), whereas the noncompacted to compacted myocardial mass ratio was the most specific, only being met in 61 (4.4%).

**Conclusions:**

A significant proportion of an asymptomatic population free from CVD satisfy all currently used cardiac magnetic resonance imaging diagnostic criteria for LVNC, suggesting that those criteria have poor specificity for LVNC, or that LVNC is an anatomical phenotype rather than a distinct cardiomyopathy.

## Central Illustration

**Figure:**
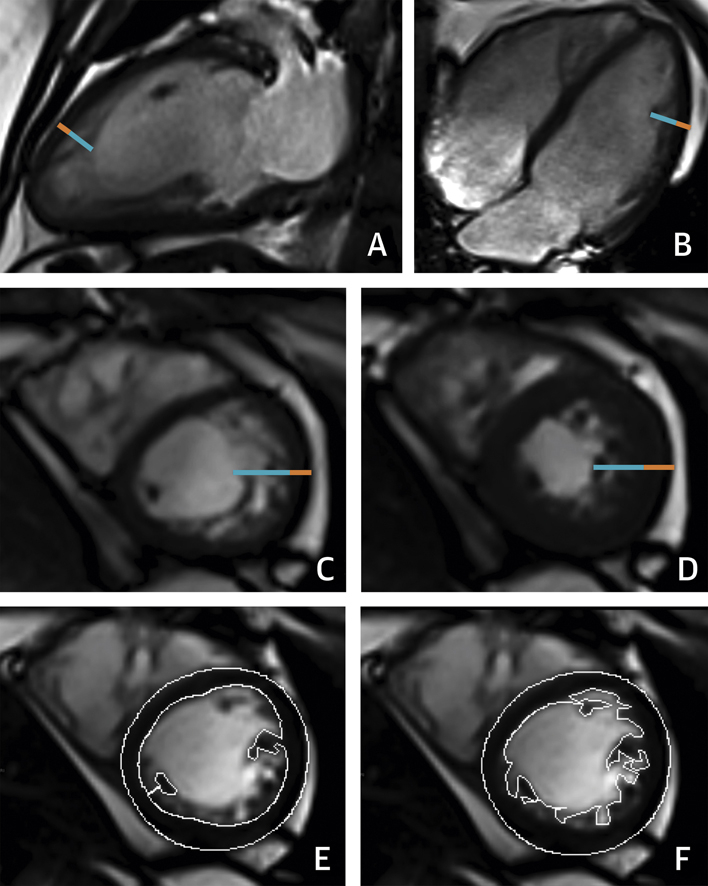

Left ventricular noncompaction (LVNC) is characterized as a primary genetic cardiomyopathy by the American Heart Association, but is characterized by the European Society of Cardiologists as an “unclassified cardiomyopathy,” aptly demonstrating some of the controversy that surrounds this condition 1, 2, 3. Previously considered a rare cardiomyopathy, there has been a rapid proliferation in publications regarding this entity, raising the question of whether this is a result of better identification of those with the disease or whether it is being over-diagnosed due to the rapid expansion in the utilization of cardiac imaging and the ever-improving visualization of cardiac structures 4, 5. More than 8% of athletes meet 1 of the 3 current echocardiographic criteria for LVNC, whereas 43% of a healthy population cohort meet the most commonly used cardiac magnetic resonance imaging (CMR) threshold for diagnosis measured on long axis cine sequences as proposed by Peterson et al. 6, 7, 8. In addition, a high prevalence of LVNC has been observed in both dilated and hypertrophic cardiomyopathies 9, 10.

Since the original CMR criteria was proposed by Petersen et al. (7), several other groups have developed alternate diagnostic criteria with improved sensitivity and specificity, utilizing measurements on both short axis systolic and diastolic views of the left ventricle as well as quantifying the compacted-to-noncompacted myocardial mass ratio 11, 12, 13. However, given the earlier findings from multiple studies utilizing multiple imaging modalities of significant noncompaction in asymptomatic cohorts free from known cardiovascular disease (CVD), it is not clear whether these new criteria help identify those with genuine disease, or whether, when applied to the general population, they will serve to further strengthen the notion of LVNC as an anatomical phenotype rather than a pathological entity. This is of significant clinical importance due to the long-term implications that currently receiving a diagnosis of LVNC entails—impacting insurance costs and necessitating long-term monitoring and follow-up. The aim of this study was to determine the prevalence of the population exhibiting LVNC, the predictors for the presence LVNC, and the physiological implications of noncompaction on cardiac function.

## Methods

### Study population

**Figure 1:**
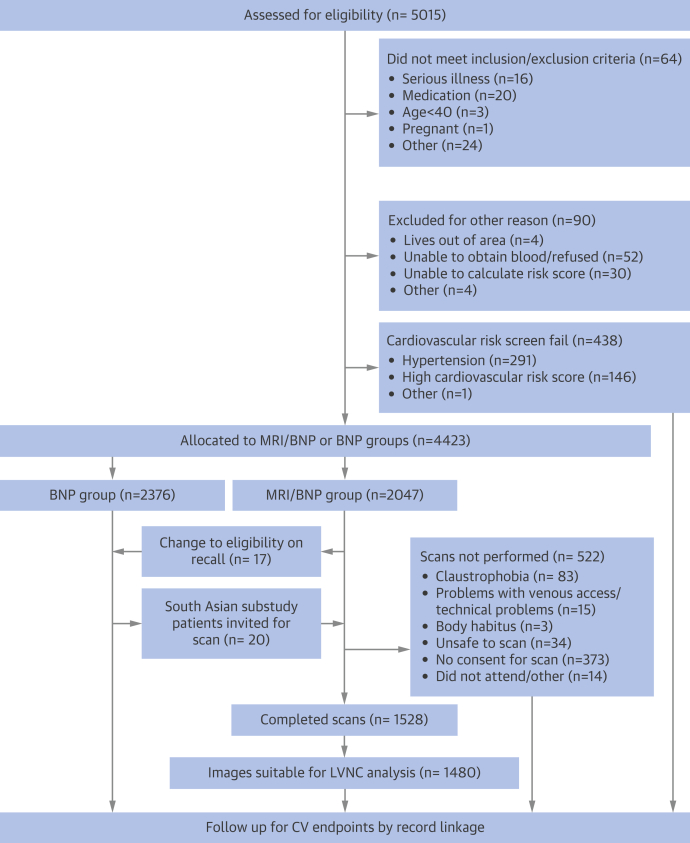
Consolidated Standards of Reporting Trials Flow Diagram of the Tayside Screening for Cardiac Events Study Diagram describes the recruitment, exclusions, final study numbers, and planned follow-up. BNP = B-type natriuretic peptide; CV = cardiovascular; LVNC = left ventricular noncompaction; MRI = magnetic resonance imaging.

Following local ethical committee approval, a cohort of 2,047 volunteers was invited to the imaging arm of the TASCFORCE (Tayside Screening for Cardiovascular Events) study. Volunteers were enrolled into the study if they: 1) were more than 40 years of age; 2) were free from CVD or other indication for statin therapy as recommended by the Scottish Intercollegiate Guidelines Network (SIGN) report 97 for “Risk Estimation and the Prevention of Cardiovascular Disease” published in February 2007; 3) had a serum B-type natriuretic peptide (BNP) level greater than their gender specific median; and 4) had a 10-year risk of coronary heart disease <20% as predicted by the Adult Treatment Panel III algorithm (14). Exclusion criteria included the following: 1) pregnancy; 2) known primary muscle disease; 3) known atherosclerotic disease—including angina, previous myocardial infarction, peripheral arterial disease, amputation, previous revascularization surgery, hypertension, heart failure, or cerebrovascular event; 4) known diabetes; 5) active liver disease; 6) other known illness or contraindication to magnetic resonance imaging (MRI); 7) participation in another clinical trial; 8) inability to give informed consent; 9) known alcohol abuse; and 10) a blood pressure >145/95 mm Hg. Details of the TASCFORCE study arms and design are encapsulated within Figure 1.

### Image acquisition

The MRI protocol has been described in detail elsewhere (15). In brief, imaging was performed using a 3-T Magnetom Trio Scanner (Siemens, Erlangen, Germany). Whole-body magnetic resonance angiography was performed using a dual-bolus injection technique with the CMR cines performed before the first contrast injection, and the late gadolinium enhancement sequences performed between the first and second contrast bolus injections. For CMR, a body matrix radiofrequency coil (6 elements) was used in combination with a spine array (up to 24 elements).

Electrocardiograph (EKG)–gated segmented breath-hold cinematic (CINE) TrueFISP (Siemens, Erlangen, Germany) images were acquired in the horizontal and vertical long axes, and in the short axis from the atrioventricular ring to the left ventricular (LV) apex using a 2-dimensional ECG-gated breath-hold segmented (CINE) TrueFISP sequence. Retrospective ECG gating was used, with 25 cardiac phases reconstructed (25 lines per segment) and 2 image slices acquired per breath-hold. Parallel imaging was also implemented (integrated parallel acquisition technique [iPAT x2]).

### Image analysis

LV mass and volume quantification was performed as previously described (15). Values were normalized to height^1.7^. For noncompaction assessment, each of the 4 diagnostic criteria was measured as follows (Central Illustration):

1. Long axis noncompaction (LAX) was measured on the horizontal and vertical LAX cine sequences, which were analyzed at end-diastole. The thickness of the compacted and noncompacted myocardium was measured at the location of maximum noncompaction as described by Petersen et al. (7). Where uncertainty existed, multiple sites were measured and the maximum noncompaction ratio recorded. An LAX noncompacted: compacted myocardial ratio ≥2.3 was considered to meet the LAX diagnosis of LVNC (7). The location of maximum noncompaction was recorded using the American Heart Association (AHA) 17-segment model of the left ventricle.
2. Short axis noncompaction (SAX) was performed using the short axis cine images, with the region of highest noncompacted myocardium to compacted myocardium ratio measured both in diastole and systole, as described by Stacey et al. (12) and Grothoff et al. (13). The apical LV (segment 17) was excluded from analysis. A diastolic SAX (SAX_DIAS_) noncompacted: compacted myocardial ratio ≥3 was considered to meet the SAX_DIAS_ diagnosis of LVNC, whereas a systolic SAX (SAX_SYST_) noncompacted:compacted myocardial ratio ≥2 was considered to meet the SAX_SYST_ diagnosis of LVNC. The location of maximum noncompaction in both systole and diastole was recorded using the AHA 17-segment model of the left ventricle.
3. Noncompacted myocardial mass (NC_MASS_) was measured as described by Jacquier et al. (11). Endocardial and epicardial contours were defined on the SAX stack at end-diastole with the papillary muscles included in the compacted mass. A new endocardial contour was then defined to incorporate the noncompacted trabeculae to calculate the global LV mass. The NC_MASS_ was calculated as the difference between the global LV mass and the compacted LV mass. A noncompacted mass >20% of the global LV mass was considered to meet the NC_MASS_ diagnosis of LVNC.

All individuals had the LAX noncompacted: compacted myocardial ratio measured, however only those with a maximum LAX noncompacted: compacted myocardial ratio ≥2 underwent SAX_SYST_, SAX_DIAST_ and NC_MASS_ measurements. A lower ratio threshold of ≥2 was chosen to widen the population sampled to ensure adequate capture of all individuals likely to meet any of the diagnostic criteria.

Those who met all 4 diagnostic criteria for LVNC were taken as demonstrating the LVNC phenotype. The Central Illustration demonstrates the measurements performed using the 4 techniques. All analysis was performed using commercial software (Argus, Siemens Multi-Modality Work Platform, version VB 15, Seimens) by 1 of 2 observers. Fifteen study datasets were read by both observers, with 1 observer reading them twice to calculate intraobserver and interobserver variability for each of the 4 measures.

### Statistical analysis

Data are expressed as mean ± SD for continuous variables, median (range) for ordinal variables and number of patients (%) for nominal variables. Normality tests were performed; if the test failed, where possible standard transformations such as square root, reciprocal, or logarithmic transforms were used to generate a Gaussian distribution. An independent sample Student *t* test was used to test the null hypothesis that samples originate from the same source. Chi-square or Fisher’s exact tests were used as appropriate to compare nominal data. Two-way mixed, absolute agreement, average measure intraclass correlation coefficients (ICC) for each of the 4 measures of noncompaction were determined with ICC >0.75 = excellent, 0.4 to 0.75 = good, <0.40 = poor, and <0.20 = slight. All data were analyzed using SPSS statistical package (version 21.0, IBM SPSS, Chicago, Illinois). Significance was adjusted for multiple comparisons using a Bonferroni correction.

## Results

Of the 1,528 volunteers who completed the imaging protocol, 48 were excluded due to inadequate image quality. This resulted in 1,480 (age 54.1 ± 8.3 years, 38% male) undergoing complete imaging with diagnostic quality images allowing measurement of all 4 measures of noncompaction.

The average maximum LAX noncompacted ratio within the entire cohort was 1.78 ± 0.63. A total of 296 (20.0%) of 1,482 analyzed datasets demonstrated an LAX ratio of ≥2 and were therefore included in subsequent analysis. Of the 296 who underwent all 4 diagnostic tests for LVNC, 219 (74%) met at least 1 diagnostic criterion for LVNC, 117 (39.5%) met 2 criteria, 63 (21.3%) met 3 criteria, and 19 (6.4%) met all 4 diagnostic criteria (Table 1). A total of 186 (62.8%) met the LAX criteria with the most common location for the maximum LAX noncompaction ratio found in the apical lateral wall. A total of 106 met the SAX_DIAST_ (35.8%) criterion, with the most common site of maximum noncompaction found in the apical lateral wall (segment 16). A total of 65 (22%) met the SAX_SYST_ criterion, with the most common site of maximum noncompaction found in the apical lateral wall (segment 16). A total of 61 (20.6%) met the NC_MASS_ criteria. Thus, 14.8% of the normal population met at least 1 of the current CMR criteria for LVNC, whereas 1.3% met all 4 of the proposed diagnostic criteria for LVNC.

Those who met all 4 of the diagnostic criteria (and were therefore considered in our study to exhibit the LVNC phenotype) demonstrated no significant differences in demographics, allometric measures, or cardiovascular risk factors (Table 2). They did however show significantly lower LV mass index (LVMI) (36.1 ± 9.2 g/m^1.7^ vs. 42.5 ± 9.5 g/m^1.7^, p = 0.004), higher LV end systolic volumes (LVESV) (20.6 ± 6.1 g/m^1.7^ vs. 17.1 ± 5.5 ml/m^1.7^; p = 0.006), lower ejection fraction (EF) (64.7 ± 9.2% vs. 69.0 ± 6.5%; p = 0.005), and lower LV mass volume ratio (LVMVR) (0.62 ± 0.10 g/ml vs. 0.79 ± 0.15 g/ml; p < 0.001) (Table 3). A significant but weak inverse correlation was seen between systolic blood pressure and the LAX noncompaction ratio (B = −0.004; p = 0.001) with an inverse correlation observed between the degree of myocardial noncompaction and LV mass (B = −0.006; p < 0.001) (Table 4).

Repeatability for the LAX measures was good for intraobserver repeated measures (ICC: 0.59; 95% confidence interval [CI]: -0.28 to 0.87), and poor for interobserver measures (ICC: 0.28; 95% CI: −1.3 to 0.76). Repeatability for the SAX_DIAS_ measures was good for intraobserver repeated measures (ICC: 0.65; 95% CI: −7.42 to 0.57), and good for interobserver measures (ICC: 0.73; 95% CI: -0.18 to 0.93). Repeatability for the SAX_SYST_ measures was only slight for intraobserver repeated measures (ICC: 0.19; 95% CI: −1.92 to 0.77), and good for interobserver measures (ICC: 0.50; 95% CI: −0.49 to 0.87). Repeatability for the NC_MASS_ measures was good for intraobserver repeated measures (ICC: 0.70; 95% CI: −0.08 to 0.92), and excellent for interobserver measures (ICC: 0.88; 95% CI: −0.51 to 0.97).

## Discussion

In our study, almost 15% of the population meet at least 1 of the current CMR diagnostic criteria for LVNC, and 1.3% of an asymptomatic population free from known CVD meet all 4 current CMR criteria. In addition, we demonstrated that the presence of LVNC is not associated with demographics, body shape, or biochemical markers of CVD.

Our findings are comparable with previous work in the MESA (Multi Ethnic Study of Atherosclerosis) population, in which an LAX noncompaction ratio >2.3 was observed in 43% of 323 participants free from cardiac disease and hypertension (8). The lower incidence in our cohort is likely due to 2 factors. The first is the additional use of the LV outflow tract LAX view of the LV in the MESA cohort, thereby increasing the likelihood of detecting a region of greater noncompaction using 3 LAX views compared with 2 views alone. The second is the multiethnic cohort examined in this previous study, since there is a greater noncompacted mass in healthy blacks compared with healthy whites 6, 16. We have thus validated the previous observations made in the MESA cohort within a second, much larger population study, and further developed and strengthened the original observations demonstrating that even when alternate or more stringent combined criteria are used, a significant proportion of the general population would still be considered to have LVNC. A significant but weak correlation was seen between systolic blood pressure and LAX noncompaction ratios, consistent with prior observations by others (17). No correlation was seen between allometric measures and noncompaction ratios, suggesting that the presence and thickness of trabeculations are not determined by body size or composition. In our study we observed that those with LVNC had a higher ESV with a lower LV mass (LVM) and EF. Previous work in the Framingham study has shown that inclusion of the trabeculae within the myocardial mass contours results in a significant increase in LVM and a decrease in LV volumes consistent with our observations (18). Thus, these findings are most likely due to the technique used for the measurements of mass, volume, and function in the current study in which trabeculations were included in the blood pool rather than within the LVM. However, follow-up of this cohort is required to confirm that this is the case and that these findings are not indicative of early pathological changes. Interestingly, although a difference in LV metrics was observed between groups when only those meeting all 4 criteria were looked at, only a very weak correlation was seen between the noncompaction ratio and LV measures when only the LAX measure was used. This suggests that LAX noncompaction is not only the least specific criterion (resulting in the most over diagnosis) in our study cohort, but also has limited implications for LV remodeling.

The observation of high incidence of LVNC in 2 separate population studies indicates 1 of 2 possibilities. One is that the current diagnostic criteria lack specificity for the accurate identification and diagnosis of LVNC, with resultant extensive over diagnosis in normal individuals. Indeed, the poor correlation between the measures, with 15% of the study cohort meeting at least 1 criterion but <2% meeting all 4, suggest that the feature they are measuring is poorly captured by any 1 of the techniques. This is in keeping with previous observations using echocardiographic diagnostic criteria in which, in a population with known heart failure and diagnosis of LVNC, only 29.8% met all 3 criteria, whereas 36.3% fulfilled only 1 criterion (19). In the current study we have not assessed the use of fractal analysis, which has been previously described to better differentiate healthy volunteers from those with pathological LVNC (20). While this holds some potential, it must be noted that the cohort in whom they demonstrated a lack of over-diagnosis was free from hypertension and nonobese. Because both of these increase trabecular complexity, its ability to differentiate a typical patient presenting with shortness of breath and both of these comorbidities from true LVNC remains to be proven. Finally, trabecular complexity using fractal analysis in both gene-negative and gene-positive hypertrophic cardiomyopathy is both within the same diagnostic range seen in LVNC. Thus, its specificity in the diagnosis of LVNC is questionable (10). One potential solution has been proposed that moves from a purely imaging-based diagnosis to a diagnosis that is more holistic and closer to that of arrhythmogenic right ventricular cardiomyopathy — requiring, in addition to meeting imaging criteria either a family member with LVNC, a regional wall motion abnormality, LVNC-related complications (arrhythmia, heart failure, or thromboembolism), or carrier status of a genetic mutation known to be associated with LVNC (21).

The second possibility is that noncompaction is an anatomical phenotype rather than a pathological cardiomyopathy. At 10-year follow-up of the aforementioned MESA study, those who met the Peterson et al. (7) criterion for LVNC demonstrated no significant difference in LVEF over the follow-up period, nor any difference in cardiovascular events compared to those who did not meet the criterion (22). Planned 5- and 10-year follow-up within our TASCFORCE study group will provide further useful information on the clinical impact of noncompaction within this population. It may simply be that those currently diagnosed with LVNC are those with the anatomical LVNC phenotype who subsequently develop dilated or hypertrophic cardiomyopathy. The argument in favor of this is strengthened by a recent study in patients with heart failure that demonstrated a lack of significant association between noncompaction ratios and subsequent major adverse cardiovascular events (9).

### Study limitations

First, we only conducted a full analysis of all diagnostic criteria in those with an LAX ratio of ≥2, and thus may have underestimated the total number who may have met 1 or more of the other 3 diagnostic criteria. However, this is only likely to further strengthen our observation of overdiagnosis if more participants without this criterion happened to meet 1 of the other 3 criteria. Second, a selection criterion for recruitment into the imaging arm of the TASCFORCE study was BNP above the gender specific median. Given the known association between BNP and heart failure, this could bias the results towards detecting a higher prevalence of a phenotype that is traditionally associated with heart failure and a poor clinical outcome. It could also increase the prevalence of those with diastolic dysfunction, which may in turn affect trabeculation quantification if there is a significant remodeling epiphenomenon component to these measures. No previous reports have described an association between these 2, nor is there any evidence of absence of association. However, of some reassurance, we did not observe any correlation between BNP and noncompaction ratios, nor were BNP levels significantly higher in those who met all 4 criteria, suggesting the impact of any potential bias of this is likely to be small.

## Conclusions

A significant proportion of an asymptomatic population free from CVD satisfy all currently used CMR diagnostic criteria for LVNC, suggesting that either these all have poor specificity for LVNC, or that LVNC is an anatomical phenotype rather than a distinct cardiomyopathy.

Perspectives **COMPETENCY IN MEDICAL KNOWLEDGE:** The current cardiac MRI criteria for diagnosis of LVNC lead to over-representation of its frequency in asymptomatic patients without other manifestations of heart disease. This suggests that LVNC is an anatomical phenotype rather than a distinct cardiomyopathy.

**TRANSLATIONAL OUTLOOK:** Further studies are needed to validate more specific, comprehensive criteria beyond simple anatomical measures on cardiac imaging that identify patients with LVNC at risk of developing adverse clinical events such as arrhythmia, heart failure, or thromboembolism.

## Figures and Tables

**Central Illustration:**
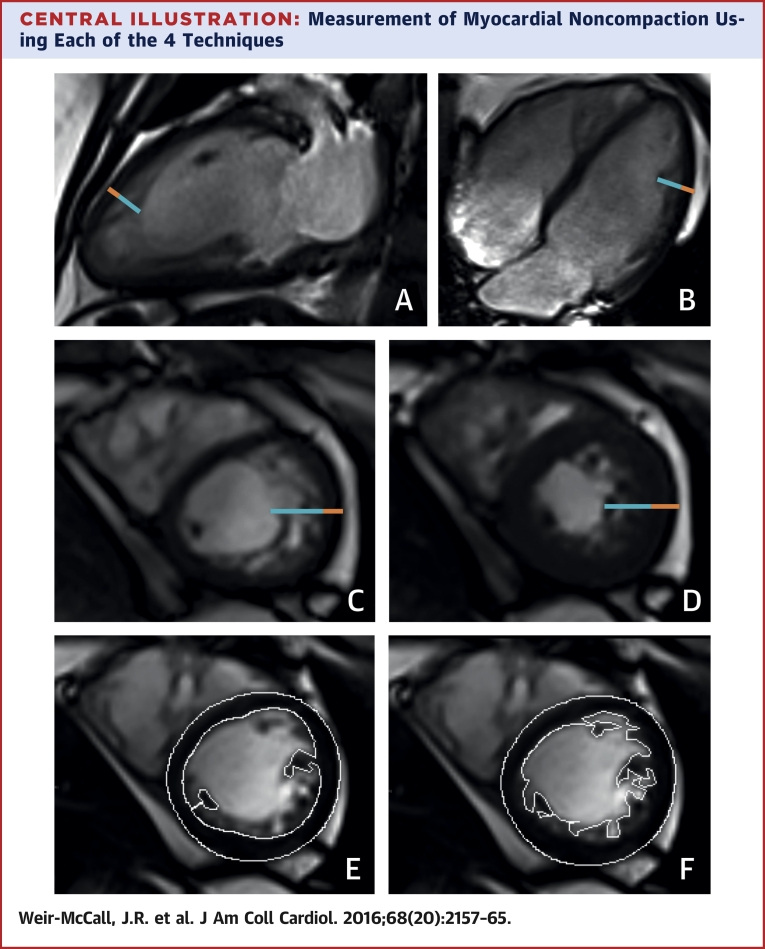
Measurement of Myocardial Noncompaction Using Each of the 4 Techniques **(A, B)** Images demonstrate long axis noncompaction ratio measurement (**orange line** = compacted myocardium, **blue line** = noncompacted myocardium) with a maximum long axis noncompaction ratio of 3.4 obtained in the anterior apical wall. **(C, D)** Images show short axis noncompaction measurements are demonstrated at diastole **(C)** where the maximum noncompaction ratio = 3.6 and systole **(D)** where the maximum noncompaction ratio = 2.2. **(E, F)** Images delineate compacted and total myocardial mass contours giving a noncompacted mass of 24% of the total mass.

**Table 1:** Breakdown of the Currently Used Cardiac Magnetic Resonance Imaging Diagnostic Criteria for Left Ventricular Noncompaction in the Whole Population and by Sex

	Long-Axis	Short-Axis Diastole	Short-Axis Systole	Noncompacted Mass	All 4 Criteria
Total (n = 1,480)	186 (12.6)	106 (7.2)	65 (4.4)	61 (4.1)	19 (1.3)
Male (n = 565)	71 (12.6)	42 (7.4)	20 (3.5)	23 (4.1)	6 (1.1)
Female (n = 915)	115 (12.6)	64 (7.0)	45 (4.9)	38 (4.2)	13 (1.4)

Values are n (%).

**Table 2:** Comparison of Cohort Characteristics Between Those Meeting 1, 2, 3, or 4 Left Ventricular Noncompaction Criteria

Criteria Met	0	1	2	3	4	p Value∗
N	1,262	102	54	44	19	
Male	480 (38)	47 (46)	19 (35)	16 (36)	6 (32)	0.64
Age, yrs	54.2 ± 8.2	53.9 ± 7.9	53.4 ± 9.0	53.5 ± 9.7	54.1 ± 8.6	0.97
Pulse, beats/min	63.4 ± 9.3	64.3 ± 16.1	62.3 ± 7.9	63.4 ± 10.7	60.3 ± 6.0	0.038
Systolic BP, mm Hg	123 ± 12	123 ± 12	120 ± 11	121 ± 11	118 ± 13	0.10
Diastolic BP, mm Hg	73 ± 9	73 ± 9	71 ± 9	70 ± 8	71 ± 9	0.44
ASSIGN 10-yr risk score	9.4 ± 6.7	8.9 ± 5.6	8.1 ± 5.5	9.4 ± 7.9	8.1 ± 5.2	0.42
Height, m	1.68 ± 0.09	1.68 ± 0.09	1.68 ± 0.08	1.70 ± 0.10	1.67 ± 0.09	0.69
Weight, kg	75.5 ± 14.5	76.1 ± 13.4	75.1 ± 14.1	75.3 ± 13.1	69.9 ± 12.1	0.09
BMI, kg/m^2^	26.8 ± 4.3	26.9 ± 3.5	26.7 ± 4.6	26.1 ± 3.7	25.0 ± 3.0	0.019
Current smoker	152 (12)	9 (9)	5 (9)	3 (7)	0 (0)	0.15
Ex smoker	328 (26)	30 (29)	15 (28)	18 (39)	5 (26)	1.00
Nonsmoker	482 (62)	61 (61)	34 (63)	23 (52)	14 (68)	0.64
Smoking pack-yrs	6.0 ± 11.7	8.2 ± 16.5	4.7 ± 9.1	4.6 ± 8.2	6.3 ± 12.9	0.90
FH of CVD	328 (26)	26 (25)	13 (24)	12 (27)	6 (32)	0.60
Total cholesterol, mmol/l	5.49 ± 0.98	5.28 ± 0.78	5.38 ± 0.85	5.60 ± 1.21	5.33 ± 0.96	0.48
LDL cholesterol, mmol/l	3.40 ± 0.88	3.27 ± 0.76	3.27 ± 0.72	3.51 ± 1.11	3.43 ± 0.81	0.88
HDL cholesterol, mmol/l	1.44 ± 0.43	1.39 ± 0.42	1.46 ± 0.38	1.43 ± 0.42	1.44 ± 0.37	0.95
Triglycerides, mmol/l	1.48 ± 0.86	1.39 ± 0.79	1.51 ± 1.01	1.51 ± 0.91	1.19 ± 0.59	0.15
Random glucose, mmol/l	5.18 ± 0.92	5.18 ± 0.69	4.99 ± 0.81	5.25 ± 0.97	5.38 ± 0.42	0.56
BNP, pg/ml	27.5 ± 15.6	24.1 ± 13.8	29.4 ± 20.5	32.8 ± 27.3	31.0 ± 23.4	0.39

Values are N, n (%) or mean ± SD. N for diagnostic criteria met is mutually exclusive, and based on the maximum number of criteria met by each study participant.

ASSIGN = assessing cardiovascular risk using Scottish Intercollegiate Guidelines Network; BMI = body mass index; BP = blood pressure; BNP = B-type natriuretic peptide; CVD = cardiovascular disease; FH = family history; HDL = high-density lipoprotein; LDL = low-density lipoprotein.

∗ p values are derived from comparing those meeting 0 criteria and those meeting all 4 criteria, with binary outcome variables compared using the Fisher exact test. Significance level set at p = 0.0025 after Bonferroni correction for multiple comparisons.

**Table 3:** Comparison of Left Ventricular Measures Between Those Meeting 1, 2, 3, or 4 Left Ventricular Noncompaction Criteria

Criteria Met	0	1	2	3	4	p Value
LVM, g/m^1.7^	42.5 ± 9.5	42.9 ± 10.2	39.8 ± 10.2	39.9 ± 8.3	36.1 ± 9.2	0.004
LVEDV, ml/m^1.7^	54.5 ± 9.7	55.8 ± 9.2	56.0 ± 12.2	57.5 ± 10.5	58.7 ± 2.7	0.069
LVESV, ml/m^1.7^	17.1 ± 5.5	17.7 ± 5.3	18.1 ± 5.4	18.6 ± 5.7	20.6 ± 6.1	0.006
LVSV, ml/m^1.7^	37.4 ± 6.5	38.1 ± 6.0	37.9 ± 8.4	38.9 ± 6.4	38.0 ± 9.7	0.78
LVEF, %	69.0 ± 6.5	68.7 ± 6.1	67.8 ± 5.5	68.1 ± 5.9	64.7 ± 9.2	0.005
LVMVR, g/ml	0.79 ± 0.15	0.77 ± 0.14	0.72 ± 0.11	0.70 ± 0.13	0.62 ± 0.10	<0.001

Values are mean ± SD.

LVEDV = left ventricular end-diastolic volume; LVEF = left ventricular ejection fraction; LVESV = left ventricular end-systolic volume; LVM = left ventricular mass; LVMVR = left ventricular mass volume ratio; LVSV = left ventricular stroke volume.

**Table 4:** Linear Regression Coefficients Change in the “Maximum Long Axis Noncompaction Ratio” Per Unit Increase in Demographic, Biochemical, and Cardiac Magnetic Resonance Imaging Measures

	B	SE	Intercept	p Value
N	1,480			
Sex	0.011	0.034	1.75	0.75
Age, yrs	−0.001	0.002	1.78	0.79
Pulse, beats/min	0.002	0.002	1.87	0.26
Systolic BP, mm Hg	−0.004	0.001	2.29	0.001
Diastolic BP, mm Hg	−0.003	0.002	1.99	0.073
ASSIGN risk score, %	−0.003	0.003	1.78	0.28
Height, m	0.12	0.18	1.55	0.50
Weight, kg	0.00	0.001	1.76	0.91
BMI, kg/m^2^	−0.002	0.004	1.81	0.60
Smoking pack-yrs	0.00	0.001	1.76	0.93
Total cholesterol, mmol/l	−0.02	0.017	1.89	0.16
LDL-cholesterol, mmol/l	−0.006	0.039	1.85	0.75
HDL-cholesterol, mmol/l	−0.06	0.02	1.78	0.11
Triglycerides, mmol/l	−0.02	0.019	1.78	0.36
Random glucose, mmol/l	0.008	0.027	1.72	0.77
BNP, pg/ml	0.000	0.001	1.75	0.95
LVM, g/m^2^	−0.006	0.002	2.02	<0.001
LVEDV, ml/m^2^	0.005	0.002	1.48	0.003
LVESV, ml/m^2^	0.008	0.003	1.63	0.012
LVSV, ml/m^2^	0.006	0.003	1.54	0.02
LVEF, %	−0.004	0.003	2.00	0.17
LVMVR, g/ml	−0.78	0.11	2.37	<0.001

B = gradient; SE = standard error; other abbreviations as in Tables 2 and 3.
